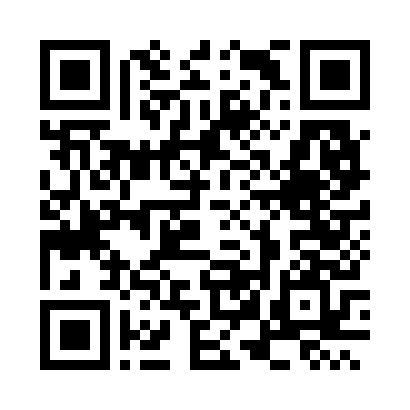# Robotic-assisted treatment of obturator nerve entrapment 5 years after retropubic tension-free vaginal tape insertion

**DOI:** 10.52054/FVVO.16.4.043

**Published:** 2024-12-27

**Authors:** H Krentel, C.D. Alt, D Andrikos, A Naem, K Otto, P Tanovska, A.S. Constantin, R.L. De Wilde

**Affiliations:** Department of Obstetrics, Gynecology, and Gynecologic Oncology, Bethesda Hospital Duisburg, Germany; Radiology Wolfgarten, Bonn, Germany; Faculty of Mathematics and Computer Science, University of Bremen, Bremen, Germany; Clinic of Gynecology, Obstetrics and Reproductive Medicine, University Hospital Saarland, Germany; Clinic of Gynecology, Obstetrics and Gynecological Oncology, University Hospital for Gynecology, Pius-Hospital Oldenburg, Medical Campus University of Oldenburg, Oldenburg, Germany

**Keywords:** Obturator nerve entrapment, retropubic TVT, robotic gynaecological surgery

## Abstract

**Background:**

Stress urinary incontinence is a frequent condition in female patients. Surgical treatment with tension-free vaginal tape (TVT) insertion is a minimally invasive option with immediate improvement of symptoms. Different possible complications have been described in the literature. Obturator nerve entrapment is a very rare complication of retropubic (rp) TVT insertion.

**Objectives:**

To show the feasibility of robotic-assisted laparoscopic mesh removal in a case of rpTVT-related entrapment of the left obturator nerve.

**Materials and Methods:**

We present the case of a 55-year-old patient who suffered from left obturator nerve dysfunction with adductor muscle atony and neuralgia after insertion of rpTVT five years earlier in an external hospital for urinary stress incontinence. We show the safety and feasibility of robotic-assisted nerve-sparing mesh removal.

**Main Outcome Measures:**

Post-interventional pain release and nerve and muscle function recovery.

**Results:**

Immediately after the surgical removal of the mesh and release of the left obturator nerve, the patient reported pain release and improvement of motoric function.

**Conclusions:**

Robotic-assisted surgery is a feasible minimally invasive alternative in the treatment of rpTVT-related obturator nerve entrapment.

## Introduction

Neurologic complications after implantation of tension-free vaginal tapes are reported more commonly after transobturatoric approach (toTVT) compared to retropubic midurethral slings (rpTVT) (Brubaker et al., 2011; Chen et al., 2024). Most of the reported neurological symptoms resolve within a few weeks postoperatively. Entrapment of the obturator nerve after rpTVT insertion is a very rare complication and is related to an excessive lateral positioning of the mesh (Baines et al., 2016). Patients report neuralgia and a motoric dysfunction of the adductor muscles. Medical treatment is usually not effective in achieving pain relief, thus a surgical intervention with partial mesh removal is often required. Reisenauer and Kraemer (2023) recently reported a case with entrapment of the right obturator nerve and mesh removal by laparotomy. Miklos et al. (2015) reported the combined vaginal and laparoscopic approach in order to release the mesh entrapment. Parikh et al. (2021) described sling removal by a combined vaginal and robotic- assisted approach. To our best knowledge, this is the first reported case of obturator nerve entrapment after rpTVT insertion treated by a robotic-assisted approach.

## Patients and Methods

We present the case of a 55-year-old postmenopausal patient who underwent rpTVT insertion for the treatment of urinary stress incontinence five years ago in an external centre for pelvic floor surgery. Immediately after surgery she complained about left hip pain, left lower extremity pain, obturator neuralgia and dysfunction of left adductor muscles.

Immediate MR imaging only revealed the suspicion of postsurgical haematoma in the left retropubic area. Medical treatment including painkillers was ineffective. The patient suffered for a period of five years from neuralgia, including constant discomfort localised in the deep pelvic floor and adductor muscle dysfunction, including limitations in walking, balancing, leg extension and hip flection. No alternative treatment approaches were recommended to the patient during this period. After five years the patient was referred to a radiologist specialising in gynaecological conditions. The MRI highlighted residuals of a possible haematoma and fibrosis close to the left obturator nerve approximately one centimetre cranial to the obturator foramen. Because of these findings and the related persisting symptoms, the patient was referred to our department and a robotic-assisted exploration and de-trapment of the left obturator nerve was recommended. During surgery we found the sling passing through the left obturator fossa causing fibrosis and adhesions around the left obturator nerve approximately one centimetre to the obturator foramen. The patient presented with a simultaneous postmenopausal tumour of the left ovary and a post-hysterectomy situation. Following presurgical transvaginal ultrasound and IOTA criteria, the ovarian lesions were diagnosed as cystadenoma. Therefore, we additionally indicated bilateral salpingectomy and unilateral oophorectomy. The patient opted for transvaginal specimen extraction to avoid a larger abdominal incision. The surgery has been carried out with the DaVinci X system (Intuitive, USA). We used 4 robotic arms, one auxiliary trocar, a 30-degree optic and bipolar and monopolar energy. The surgery began with mobilisation of the adherent sigmoid colon and identification of the left infundibulopelvic ligament. The left ovary and fallopian tube were then mobilised from the pelvic sidewall and the left ureter was identified. We excised the right fallopian tube and preserved the normal right ovary. We identified the left obliterated umbilical artery, and the external iliac vessels and opened the obturator fossa. After identification of the left obturator nerve, we followed the nerve to identify the fibrotic lesion close to the obturator foramen. The 4th robotic arm was used to open the retroperitoneal pace by moving the obliterated umbilical artery. After identifying the rpTVT mesh, we partially removed it by releasing the nerve from its fibrotic entrapment. Smaller branches of the obturator vessels were ligated, but the main obturator vessels were preserved. All specimens were extracted through a small colpotomy at the end of the surgery.

## Results

The surgery was carried out without any intraoperative and postoperative complications.

The surgical time was 2 hours and 18 minutes. The blood loss was less than 100 ml. Postsurgical recovery was fast, and the patient reported a complete improvement of obturator neuralgia and discomfort on the first postsurgical day. Additionally, the motoric nerve function was immediately improved with a return of the adductor muscle function in the following weeks after mesh removal.

## Discussion

Obturator nerve entrapment after rpTVT implantation is a very rare complication causing severe symptoms such as obturator neuralgia, pelvic discomfort and hip pain, lower extremity pain and motoric dysfunction of adductor muscles. In most reported cases the nerve is entrapped and adherent to the mesh material. Imaging does not easily reveal the erroneous position of the sling but shows the suspicion of haematoma and fibrosis.

However, in all published cases patients reported typical symptoms, which should point clinicians towards a TVT-related complication when these symptoms occur immediately after mesh insertion. The immediate surgical removal of the sling within the first 5-7 days after insertion would be the easiest way of treatment of this rare complication. As symptoms related to obturator nerve entrapment after rpTVT insertion won´t improve over time, the surgical (partial) removal of the mesh is also the treatment of choice weeks or years after the initial surgery. The robotic-assisted technique with 4 arms allows for a minimally invasive approach to the deep retroperitoneal pelvic localisation of the nerve entrapment. While one arm is moving the obliterated umbilical artery medially, the bipolar and monopolar instruments in combination with a 30-degree scope can easily reach the deep retropubic surgical field due to its possible angulations and the extremely precise visualisation and movements.

This case highlights the rare possibility of obturator nerve entrapment after rpTVT insertion and shows the safety and feasibility of a robotic-assisted approach.

## Conclusions

Robotic-assisted laparoscopic surgery is a feasible minimally invasive alternative in the treatment of rpTVT-related obturator nerve entrapment. This rare complication should be considered when patients present with typical symptoms immediately following a sling implantation.

## Video scan (read QR)

https://vimeo.com/995013628/ccb65dcf22?share=copy

**Figure:**